# Regulatory T-cells in Reproductive Medicine: Implications for Endometriosis-Associated Infertility and Implantation Immunity

**DOI:** 10.7759/cureus.98839

**Published:** 2025-12-09

**Authors:** Sagiri Taguchi, Terumi Hayashi

**Affiliations:** 1 In Vitro Fertilization (IVF) Center, Oak Clinic, Osaka, JPN

**Keywords:** cell therapy, endometriosis, immune tolerance, implantation, low‑dose il‑2, recurrent implantation failure, regulatory t-cells

## Abstract

Regulatory T-cells (Tregs) are central to peripheral immune tolerance and act as key players that sustain the immune homeostasis required for embryo receptivity, implantation, and placentation. Treg dysfunction accelerates inflammation, angiogenesis, and lesion progression in endometriosis, whereas preclinical work indicates that adoptive Treg supplementation can restrain the advancement of endometriosis‑like lesions. Using the 2025 Nobel Prize in Physiology or Medicine scientific background as a starting point, this review summarizes Treg basic biology, roles in reproductive immunology, the pathophysiology of immune‑mediated infertility and endometriosis, and therapeutic prospects centered on Treg induction and supplementation.

## Introduction and background

The 2025 Nobel Prize in Physiology or Medicine recognized the foundational significance of identifying CD4⁺CD25⁺FOXP3⁺ regulatory T-cells and elucidating the mechanisms of immune tolerance, reshaping our understanding of self‑tolerance, autoimmunity, and tumor immunity [1]. The accompanying scientific background systematically positions the principle that Tregs preserve tissue homeostasis by controlling self‑reactive responses [1], and this principle directly extends to reproductive phenomena that require maternal-fetal immune tolerance [1-3].

## Review

Physiological roles of Tregs in reproductive immunology

**Figure 1:**
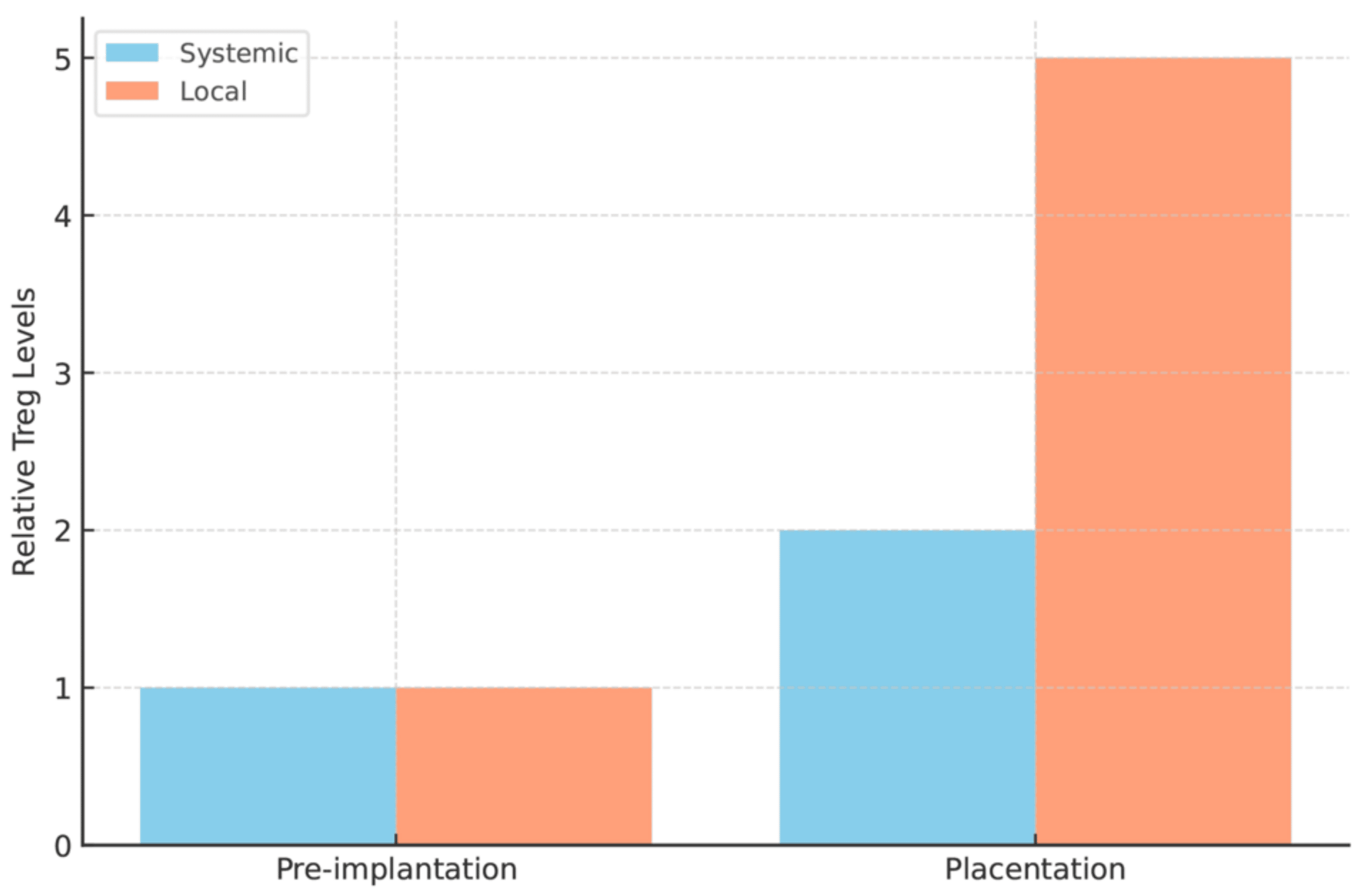
Systemic vs. local Treg dynamics at preimplantation vs. placentation A conceptual bar graph illustrates the relative abundance of regulatory T-cells (Tregs) in the blood (systemic) compared to the uterus/decidua (local) at two key timepoints in early pregnancy: preimplantation (before embryo implantation) and placentation (when the placenta is establishing). At preimplantation, Treg levels are low both systemically and in the endometrium (baseline levels). By placentation, local Tregs (orange bars) in the decidua increase dramatically, reflecting their crucial role in promoting tolerance at the maternal-fetal interface. Systemic Tregs (blue bars) also rise modestly by placentation, but to a far lesser extent than local Tregs. The bar heights represent relative Treg quantities (arbitrary units), highlighting that decidual Tregs expand substantially with the progressing pregnancy, whereas circulating Tregs show a mild increase. All labels are clear (no overlap or cutoff text), and a legend distinguishes systemic vs. local Tregs Image credit: This is an original image created by the authors Sagiri Taguchi and Terumi Hayashi

Because the mother must immunologically accept a fetus bearing semiallogeneic antigens, pregnancy features systemic and local increases in Tregs that support immune tolerance [2-4]. In mice, depletion of Tregs during the peri-implantation and early gestational windows causes implantation failure and increases embryo resorption, whereas their requirement for late gestational maintenance appears lower, indicating stage specificity [3]. Integrating evidence from humans and mice, Tregs support embryo invasion and placentation by limiting inflammation, suppressing effector T-cells, and modulating uterine artery remodeling and angiogenesis [4,5]. Qualitative differences among Treg subsets, such as peripherally induced Tregs (pTregs) and activated/effector Tregs, likely condition the peri-implantation immune milieu and influence pregnancy outcomes [4,5] (Figure 1).

Immunologic infertility and Tregs

In immune‑mediated infertility, including recurrent implantation failure and recurrent miscarriage, disruption of the Th1/Th2/Th17/Treg balance, together with quantitative reductions and qualitative dysfunction of Tregs, has been reported and is considered a key immunologic substrate of poor prognosis [4,6]. An appropriate Treg response in the early peri-implantation period promotes fetal acceptance, restrains excessive inflammation, and facilitates vascular remodeling, potentially influencing embryo transfer outcomes in assisted reproductive technologies (ARTs/in vitro fertilization) [4,5].

Endometriosis: Treg dysfunction and opportunities for disease modification

**Figure 2:**
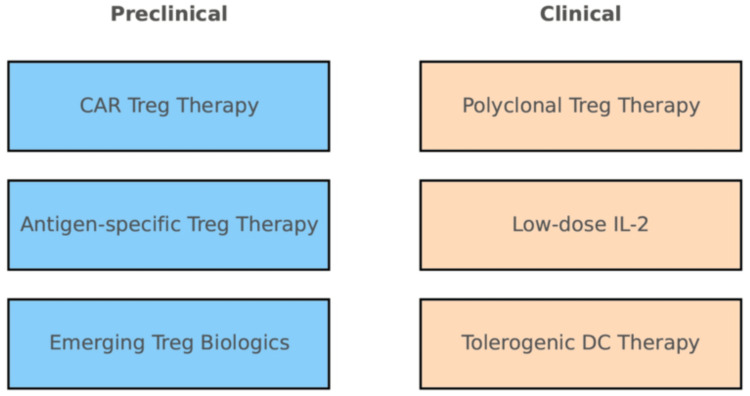
Preclinical vs. clinical-stage Treg therapies This two-panel schematic separates Treg-directed therapies by their development status. The left panel (blue shaded) lists approaches remaining in preclinical research (no human trials yet), such as CAR Treg therapy, antigen-specific Treg therapies, and other emerging Treg-based treatments. The right panel (peach shaded) lists therapies that have entered clinical trials or clinical use, including polyclonal Treg therapy (adoptive Treg transfer tested in humans), low-dose IL-2 therapy (in clinical trials for autoimmune diseases), and tolerogenic DC therapy (tolerogenic dendritic cell vaccines tested in early trials). The two sections are distinctly colored to emphasize the division. All text labels are legible, with no truncation, facilitating a clear side-by-side comparison of therapies by stage of translation CAR: chimeric antigen receptor; IL-2: interleukin 2; DC: dendritic cell Image credit: This is an original image created by the authors Sagiri Taguchi and Terumi Hayashi

Endometriosis is a disease in which ectopic endometrial tissue engrafts, proliferates, and progresses within a chronic inflammatory microenvironment; immune dysregulation is central to its pathophysiology [7]. In mouse models, Treg dysfunction (including experimental depletion) enhances local inflammation and angiogenesis and worsens the progression of endometriosis‑like lesions [7]. This supports a cascade of Treg breakdown, which contributes to heightened inflammation/angiogenesis resulting in lesion expansion [8]. Recent preclinical work further demonstrates that adoptive transfer (supplementation) of Tregs significantly suppresses the progression of endometriosis‑like lesions in Treg‑depleted mice, suggesting the feasibility of disease‑modifying intervention [9]. In nonhuman primates and humans, quantitative and functional Treg alterations have also been observed in association with endometriosis, bolstering the plausibility of clinical translation (Figure 2) [8].

Toward application in assisted reproductive medicine

Tregs As Biomarkers

Quantifying Treg abundance, phenotype, and function in peri-implantation endometrium or peripheral blood may enable stratification of recurrent implantation failure, individualized optimization of embryo transfer timing, and perioperative/peritransfer immunomonitoring [4,5].

Therapeutic Concepts Targeting Tregs

**Figure 3:**
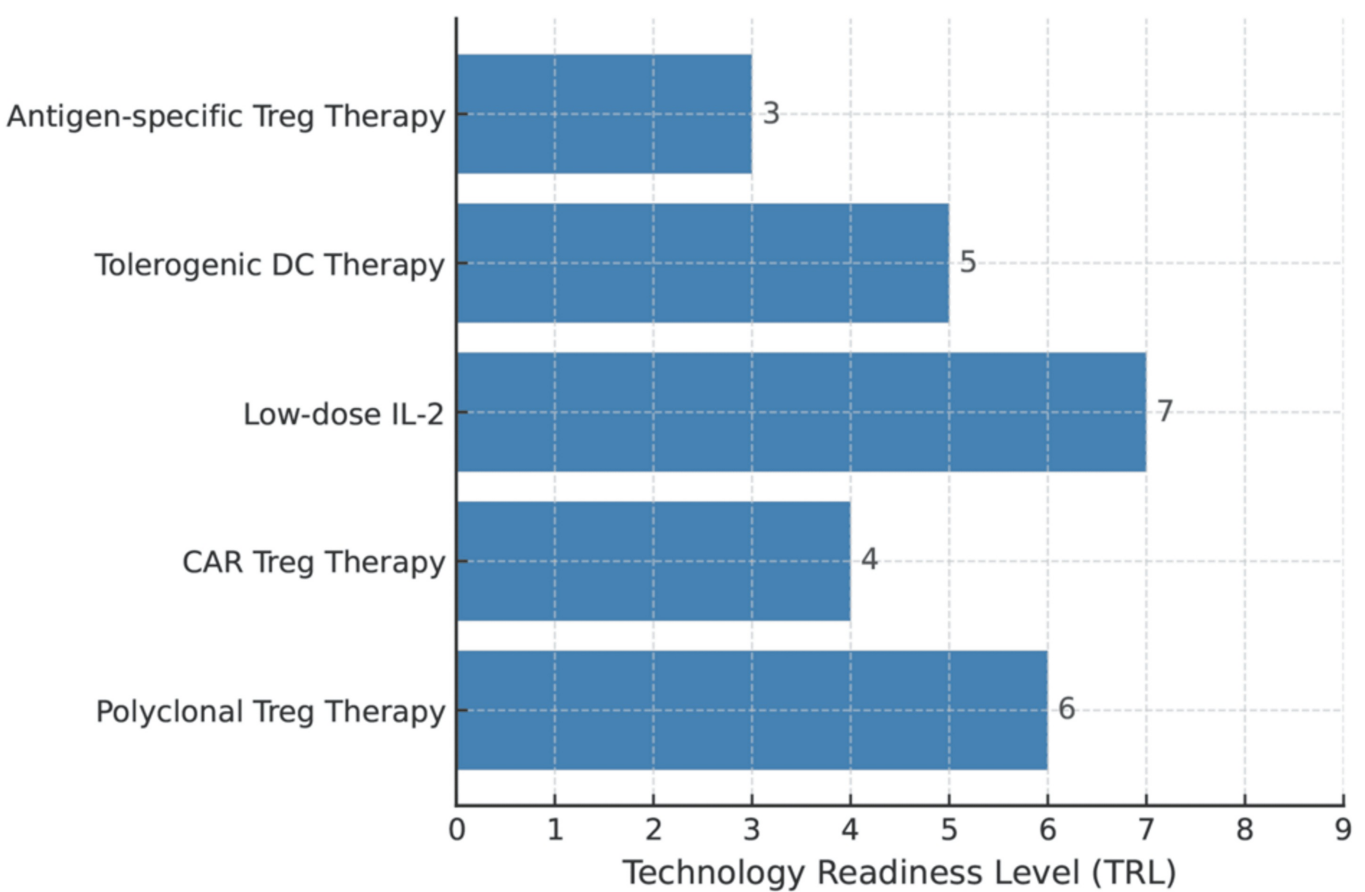
Transthyretin-like protein family mapping of Treg-based therapies A horizontal bar chart aligns various Treg-based therapeutic strategies with their approximate TRL (1-9). Each bar extends to the current readiness level of the approach (number at bar end). For example, polyclonal Treg therapy (expanded natural Tregs) is at an advanced preclinical/early clinical stage (around TRL 6), while CAR Treg therapy is earlier in development (around TRL 4). Strategies like low-dose IL-2 therapy (which expands Tregs in vivo) have reached clinical trials (around TRL 7), whereas tolerogenic DC therapy is in mid-development (TRL 5). Antigen-specific Treg therapies (e.g., tolerogenic vaccines) are still largely in proof-of-concept stages (TRL 3). All labels are clear and unbroken. The chart provides a comparative overview of how far along each Treg-based therapy is on the path from basic research to clinical application TRLs: technology readiness levels; CAR: chimeric antigen receptor; DC: dendritic cell Image credit: This is an original image created by the authors Sagiri Taguchi and Terumi Hayashi

Low‑dose interleukin‑2 (LD‑IL‑2) leverages the high‑affinity IL‑2 receptor (CD25) on Tregs to preferentially expand them; in graft‑versus‑host disease and autoimmune settings, restoration of Treg homeostasis with an acceptable safety profile has been demonstrated [10-12]. IL‑2 derivatives/complexes and other Treg‑biased agents are advancing clinically, offering scope for application to reproductive immunology alongside optimized dosing and delivery strategies [11,12]. Treg supplementation (adoptive transfer) has achieved proof of concept for attenuating lesion progression in endometriosis models and could be explored as pre‑ART conditioning or as an adjuvant to prevent postsurgical recurrence (Figure 3) [9].

Pregnancy‑Acquired Memory Tregs and Preconditioning

Pregnancy‑acquired, fetus‑antigen-specific “memory” Tregs have been reported to improve outcomes in subsequent gestations, supporting the concept of educational shaping of the Treg repertoire [13]. In ART, preconditioning (priming) of Tregs via the hormonal milieu and embryo- or seminal-plasma-derived signals, potentially combined with LD‑IL‑2, may synergize to induce and maintain memory Tregs [10-13].

Challenges for research and clinical implementation

Standardization

Harmonization of phenotypic and functional Treg assays in peripheral blood and endometrium, with prospective linkage to pregnancy outcomes, is required [4-6].

Stratified Trials

Stepwise evaluation of LD‑IL‑2 dosing, safety, and efficacy in cohorts identified with Treg deficiency/dysfunction is needed [10-12].

Bridging for Cell Therapy

**Figure 4:**
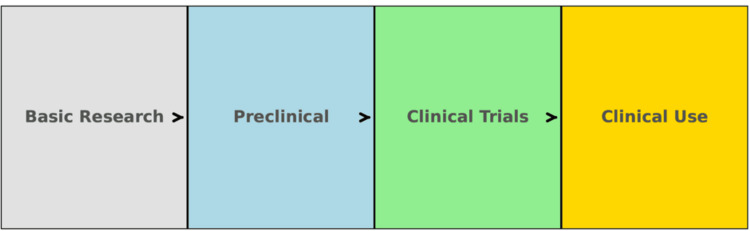
Translational roadmap Developmental roadmap for Treg therapies. A schematic timeline depicts the phases of translation for Treg-based therapies, from inception to clinical practice. Four labeled stages are shown as boxes with arrows indicating the forward progression: Basic Research → Preclinical → Clinical Trials → Clinical Use. Basic Research denotes fundamental lab studies (e.g., mechanistic discoveries); preclinical involves animal models and safety/toxicity studies; Clinical Trials are the phased human trials (Phase I/II/III) testing safety and efficacy; clinical use represents approved therapies in routine medical practice. Bold black arrows connect each stage, visually indicating the flow from one stage to the next. The layout is clean and linear, with each stage clearly boxed and labeled (black text on white background) and no crowding or truncated text. This roadmap summarizes the stepwise progression required to bring Treg therapies from the lab bench to patient care in the clinic Image credit: This is an original image created by the authors Sagiri Taguchi and Terumi Hayashi

Treg supplementation in endometriosis has preclinical proof of concept; determining optimal timing in humans (perioperative/peritransfer) and maintenance strategies (e.g., LD‑IL‑2 co‑administration) will be pivotal (Table 1) [9-12]. Trial designs must incorporate maternal-fetal safety monitoring, infection‑risk management, and evaluation of fetal development (Figure 4) [10-12].

**Table 1:** Development roadmap for Treg-targeted interventions in reproductive medicine IL-2: interleukin-2; LD-IL-2: low-dose interleukin-2; GVHD: graft-versus-host disease; AUC: area under the curve

Phase/use	Candidate intervention	Primary objective	Mechanism/intent	Primary endpoints	Existing level of evidence	Major risks/considerations	Intended population
Preclinical → early clinical	LD-IL-2	Selective expansion/activation of Tregs	Increase in CD25-high Tregs; restoration of inflammatory balance	Treg frequency/phenotype; endometrial inflammation markers; implantation rate	Clinical trial data in GVHD/autoimmunity [10-12]	Fetal safety; excessive immunosuppression	Recurrent implantation failure; immunologic infertility
Preclinical → translational bridging	Treg cell supplementation (adoptive transfer)	Disease modification and microenvironment normalization	Direct Treg supplementation to suppress inflammation and angiogenesis	Lesion volume; pain indices; recurrence rate	Proof of concept in endometriosis models [9]	Persistence of supplemented cells; timing of transfer; immunologic safety	Endometriosis (high postsurgical-recurrence risk)
Combination strategy	LD-IL-2 + Treg supplementation	Maintain Treg engraftment and function	Posttransfer maintenance/expansion of Tregs	Long-term persistence; safety	Theoretical/adjacent-field underpinnings [10-12]	Dosing design for combination therapy	Endometriosis with concomitant infertility
Diagnostic adjunct	Treg indicator panel	Stratification and timing optimization	Quantitative/functional/transcriptional assessment of Tregs in endometrium/blood	Prognostic AUC; clinical utility	Reviews and small studies [4-6,8]	Assay standardization; reproducibility	Recurrent implantation failure; prior miscarriage

## Conclusions

Tregs underpin the continuum from implantation to placentation through immune tolerance, and their significance as both mechanism and therapeutic target is increasingly established in immunologic infertility and endometriosis. The facts that Treg dysfunction exacerbates endometriosis and that Treg supplementation can suppress lesion progression strongly argue for the feasibility of immune interventions in reproductive medicine. Principles of immune tolerance highlighted by the Nobel scientific background map directly onto clinical trial designs, emphasizing stratification, optimal timing, and rational combinations.
